# Endoscopic removal of a bullet from the parapharyngeal space just anterior-lateral to the first vertebral body: case report

**DOI:** 10.11604/pamj.2022.42.132.33344

**Published:** 2022-06-17

**Authors:** Wardeh Alhusban, Qais Aljfout, Yousef Alsardia, Nabeela Tawalbeh, Mohammad Aboroman, Amer Abu-ain

**Affiliations:** 1 Department of Otolaryngology, Royal Medical Services, King Hussein Medical Centre, Amman, Jordan

**Keywords:** Bullet injury, navigation, fluoroscopy, trans-nasal endoscopic removal, case report

## Abstract

In all head and neck bullet injuries, treatment has to be individualized, as there is no universal protocol for all cases. Five important steps of management should be taken: securing airways, hemorrhage control, recognizing other injuries, foreign body removal when necessary, and repair of facial wounds. In this report, the case of a 28-year-old male patient will be presented and discussed. The patient was referred from a surgery clinic to an ear, nose and throat (ENT) outpatient clinic complaining of right neck pain, throat pain, and foreign body sensation in the throat for 2 months following a firearm injury to his face. The entry wound was observed on the left alar cartilage of his nose, which was almost healed and had left a scar. A sinus computed tomography scan showed a bullet in the right parapharyngeal space about 3 mm in front of the first vertebral body. The bullet was successfully removed using a trans-nasal endoscopic approach with the support of an image-guided navigational system and frequent fluoroscopy. These 2 methods help to replace the old traditional open approaches.

## Introduction

Bullet wounds are divided into two categories according to their velocity - high or low velocity. In general, low-velocity bullets are those traveling 1200 feet/s or less and high-velocity bullets are those that have a velocity greater than 1200 feets. The amount of surrounding soft tissue injury from a bullet wound is linked to the mass and velocity of the bullet (kinetic energy equals 1/2 mv2). Low-velocity bullet wounds cause restricted injury around the bullet path and result in minimal soft tissue damage and bone loss [1]. High-velocity injuries from rifles or a close-range shotgun blast cause significant damage to the tissue where the bullet has lodged and along the bullet path (primary cavity). These types of injuries can also cause remarkable soft tissue damage and bone loss [1]. Removal of bullets lodged in the nasopharyngeal space is complicated due to the complexity of the nasopharyngeal anatomy and its proximity to vital structures such as the base of the skull, cervical vertebrae, and the internal carotid artery [2]. Therefore, it is hard to remove the bullet safely. The classical treatment of bullet wounds involves initial debridement of the wounds and closure of soft tissue with no replacement of lost bone fragments. This may result in scar contracture and substandard functional and cosmetic end results. Over the past 10 years, the treatment has been changed towards prompt reconstruction rather than the classical delayed reconstruction. This change has been driven by the increased use of and accessibility to vascularized free tissue transfer, which allows the restoration of the soft tissue and bony structure while decreasing the formation of scar contracture [1,3]. In general, lodged missiles in the parapharyngeal space are rare [4]. This is a rare case report of a young man who walked into the Ear Nose and Throat (ENT) clinic with a bullet lodged in the parapharyngeal space parallel to the nasopharynx, anterior-lateral to the first vertebral body, for which he underwent a safe nasal endoscopic removal.

## Patient and observation

**Patient information:** in September 2019, a 28-year-old medically free, surgically free patient presented with a bullet injury to his face sustained in a fight. The bullet, fired from an unknown distance, entered the left side of his nose near his left alar cartilage and did not exist. The patient was fully conscious and well oriented as to time, person, and place upon hospital admission. He sought emergency treatment at his hometown hospital, where computed tomography revealed that the bullet was lodged in the nasopharynx exactly at the right parapharyngeal space, just 3 mm anterior-lateral to the first vertebral body. At initial presentation, the patient had minimal bleeding, which resolved spontaneously, and a cut wound at the site of entry, which was left for secondary intention. The patient was admitted for 1 day. After 3 months, the patient was referred to the ENT clinic due to throat and neck pain and foreign body sensation in the throat.

**Figure 1:**
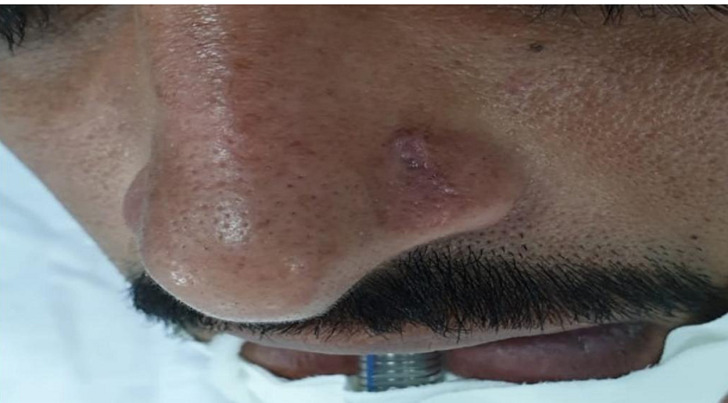
profile view of the patient

**Figure 2:**
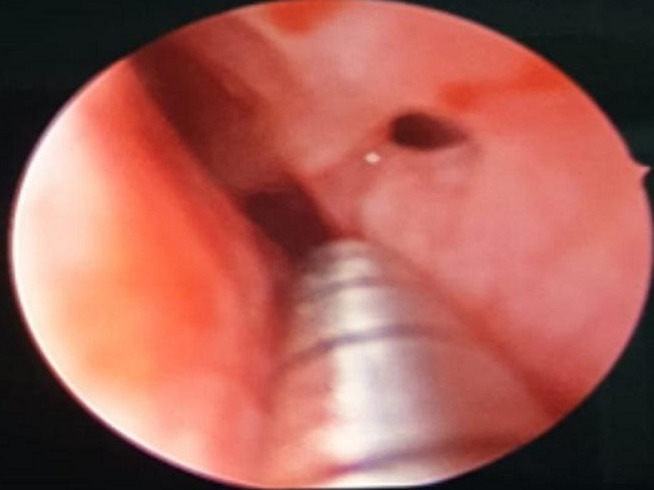
endoscopic view of the nose

**Clinical findings:** on examination, the patient had a scar on the left side of his nose (Figure 1), posterior small septal perforation (Figure 2), and adhesion between the septum and right inferior turbinate. There was no external nasal deformity or swelling.

**Timeline:** the patient presented with a bullet injury in September 2019 for which he was managed conservatively. After 3 months, he was referred to the ENT clinic and trans-nasal endoscopic removal of the missile was performed under general anesthesia on December 10, 2019. The patient recovered uneventfully and was discharged home 2 days after the surgery.

**Figure 3:**
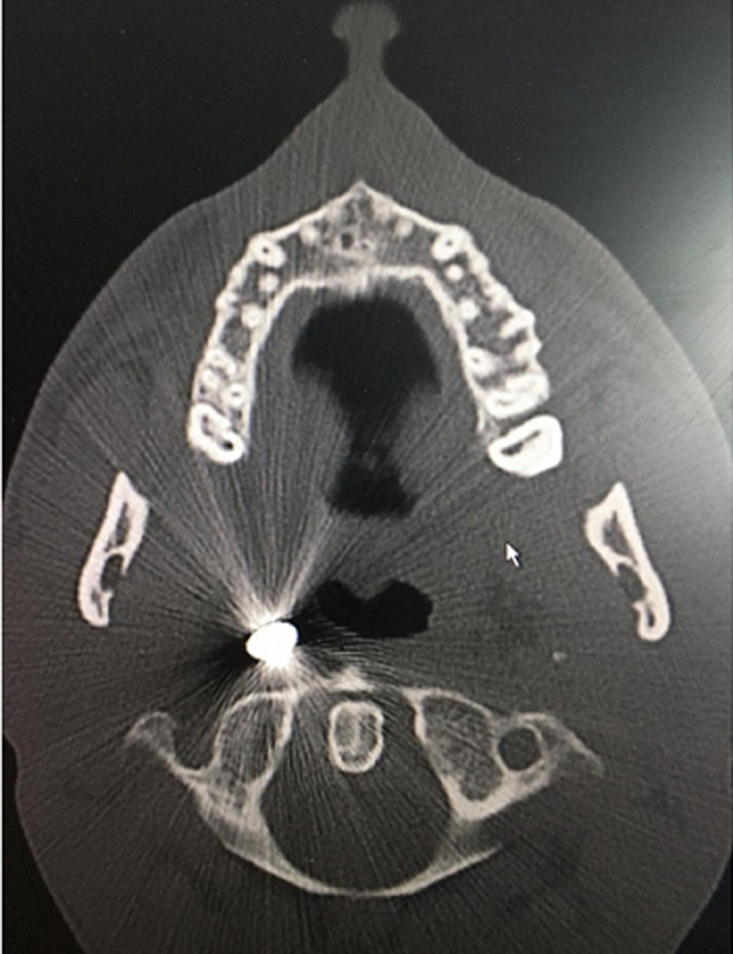
axial view of the computed tomography scan

**Figure 4:**
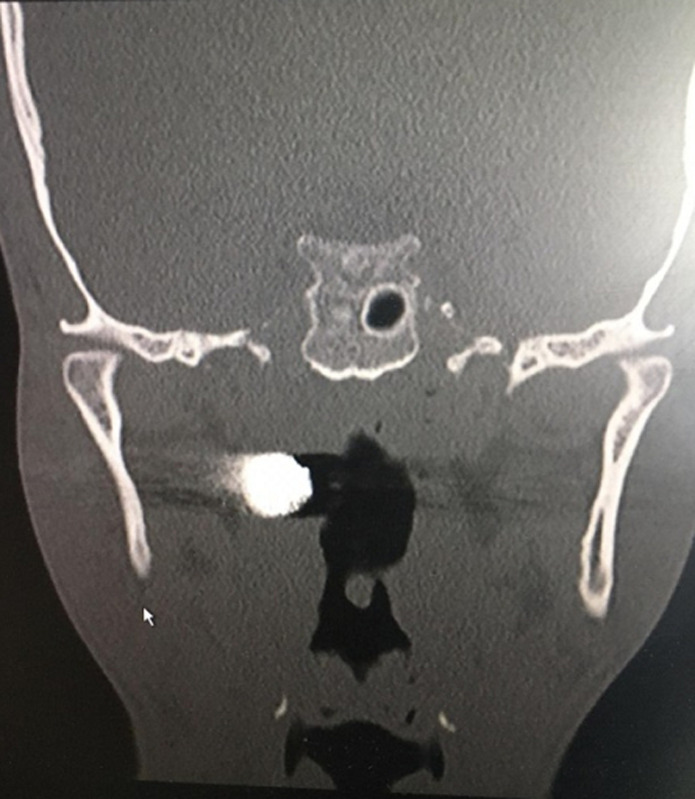
coronal view of the computed tomography scan

**Figure 5:**
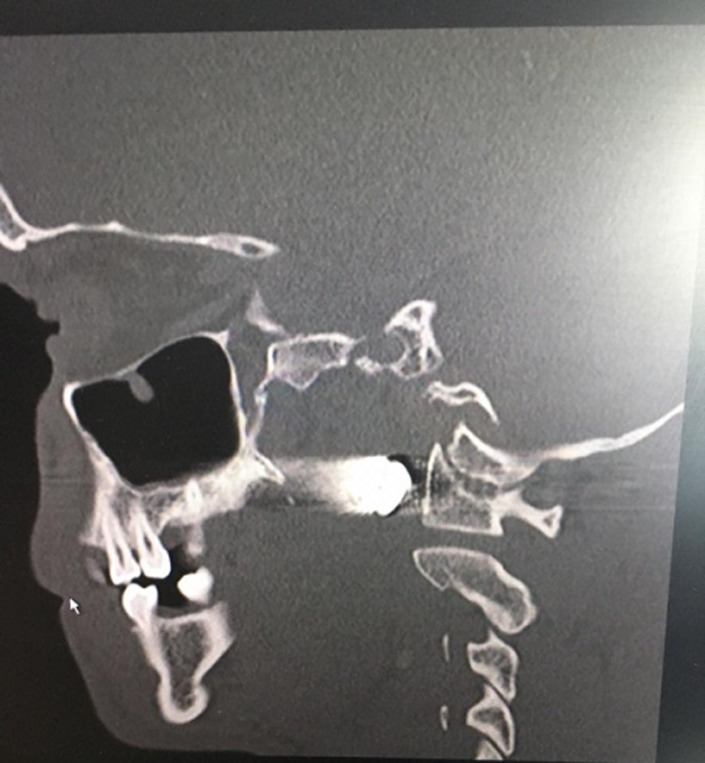
sagittal view of the computed tomography scan

**Diagnostic workup:** the computed tomography scan showed a bullet lodged in the right parapharyngeal space just 3 mm anterior-lateral to the first vertebral body as shown by the reflection of the bullet on the X-rays (Figure 3, Figure 4, and Figure 5).

**Figure 6:**
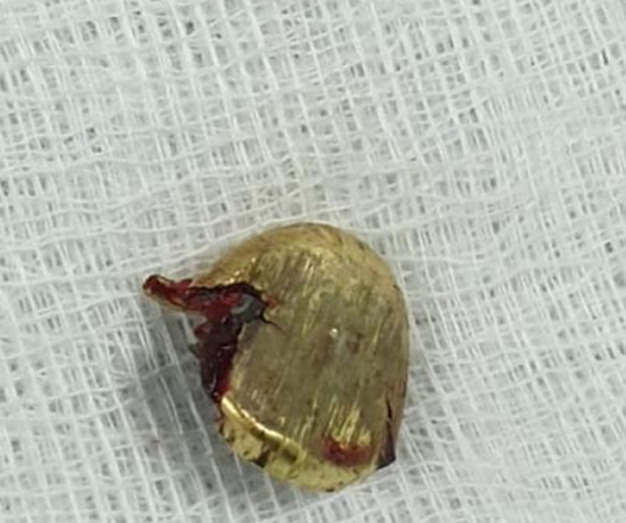
removed bullet

**Therapeutic intervention:** in December 2019, trans-nasal endoscopic removal of the bullet was performed with no complications by an ENT surgeon using a navigation protocol and fluoroscopy. At the end of the operation, bilateral nasal packs were applied. The bullet´s size was 1cmx1cm (Figure 6).

**Follow-up and outcome:** the patient recovered well in the hospital, the packs stayed on for 24 hours, and he was discharged home after 2 days of monitoring. During hospitalization, there was no evidence of cerebrospinal fluid (CSF 0leak or epistaxis).

## Discussion

Bullet injuries in the craniofacial region may cause damage to vital structures, causing irreversible, uncompensated loss and fatal consequences. If a missile penetrates the head, the bullet´s path is unpredictable, as it can go in any direction and lodge in any area of the skull [5]. The site of the foreign body tailors the approach to its removal. Sometimes the site is so critical that it contraindicates surgery, as the risks of removal would outweigh the benefits. The decision to remove a bullet in the craniofacial region depends on its location, the surgeon´s experience and skill, and the facilities available [5]. The most common area for foreign objects to be trapped in the face is the paranasal sinuses [5]. The presented case is one of the rare cases of a bullet that lodged in the parapharyngeal space at the level of the postnasal space just 3 mm anterior-lateral to the first vertebral body. Operating in this space requires consideration of the position of the internal carotid artery, which is usually located lateral to the Rosenmüller fossa [6]. The conventional open method of bullet removal in the parapharyngeal space leads to a raised morbidity rate, scarring, disfigurement, and other complications. Also, blind vigorous dissection around the bullet site without intraoperative imaging could lead to deeper migration of the foreign body. For this patient, the initial plan was trans-nasal endoscopic removal of the bullet with the use of a navigation system. Our backup option was to use a C-arm fluoroscopy (X-ray) machine. Surgery started as organized with the help of the navigation system.

During the surgery, complete healing of the postnasal wound was noted, as the operation was 3 months after the initial injury. The navigation system could not assess the changes of the bullet´s location during the procedure. Therefore, we decided to use the C-arm fluoroscopy machine to see the bullet´s location in real-time, and eventually, the bullet was removed through a small wound in the nasopharynx. In summary, the bullet was successfully removed by a trans-nasal endoscopic system approach guided by a navigation system linked with a computed tomography scanner and fluoroscopy. The navigation protocol was very useful to locate the vital structures and limit possible complications, and the use of fluoroscopy provided the exact dynamic location of the bullet [7]. The combination of the two technologies (navigation system and fluoroscopy) helped to pinpoint the actual site of the missile and provided a roadmap for precise and safe endoscopic removal [5]. This method decreases morbidity, scarring, and the duration of the surgery and enhances post-operation recovery. However, the problem here is an increased risk of radiation exposure to the surgeon, patient, and theatre staff associated with the use of fluoroscopy. The other possible procedure for this case was the trans-oral palatal split option, but it was ruled out due to the higher risk of complications, especially velopharyngeal insufficiency, dysphonia, and dysphagia [8]. The chosen approach led to a short operative time and post-operative stay and resulted in a significant reduction of infection risk and other complications.

## Conclusion

The trans-nasal endoscopic approach to remove a bullet lodged in the craniofacial area is highly recommended as long as the bullet is approachable through the endoscope. The use of a navigation system and fluoroscopy to locate the exact dynamic location of the bullet leads to a successful trans-nasal endoscopic removal approach and opens the door to replacing the traditional open surgical approach.
